# Supplementary material for: Active site specificity profiling datasets of matrix metalloproteinases (MMPs) 1, 2, 3, 7, 8, 9, 12, 13 and 14
Source: Data Brief. 2016 Feb 22;7:299–310. doi: 10.1016/j.dib.2016.02.036 (PMC4777984; doi:10.1016/j.dib.2016.02.036)
Supplement: Supplementary file 10 — Supplementary material [file mmc10.zip › WebPICS_hMMP7_G_1%/P1.html]

 

PICS results


|  |  |
| --- | --- |
| **P1\_A**  18 in 163 sites   11.0 %    effects > 10 perc. pnts.  (vice-versa in brackets)  P2prime\_T: 12.4 (14.0) |  |
| **P1\_C**  4 in 163 sites   2.5 %    effects > 10 perc. pnts.  (vice-versa in brackets)  P2\_H: 21.9 (17.5)   P2prime\_L: 62.7 (12.5) |  |
| **P1\_G**  12 in 163 sites   7.4 %    effects > 10 perc. pnts.  (vice-versa in brackets)  P3\_P: 15.2 (11.4)   P2\_N: 25.3 (23.4)   P2prime\_R: 15.8 (12.6)   P2prime\_T: 15.2 (11.4)   P3prime\_S: 18.3 (19.9) |  |
| **P1\_H**  5 in 163 sites   3.1 %    effects > 10 perc. pnts.  (vice-versa in brackets)  P2\_M: 16.9 (16.9)   P1prime\_M: 35.1 (21.9) |  |
| **P1\_K**  17 in 163 sites   10.4 %    effects > 10 perc. pnts.  (vice-versa in brackets)  P3\_V: 14.7 (10.4)   P1prime\_I: 26.9 (13.8)   P2prime\_R: 14.3 (16.3)   P3prime\_Q: 10.9 (16.9) |  |
| **P1\_N**  12 in 163 sites   7.4 %    effects > 10 perc. pnts.  (vice-versa in brackets)  P2prime\_I: 15.2 (11.4)   P3prime\_N: 11.8 (17.6) |  |
| **P1\_P**  10 in 163 sites   6.1 %    effects > 10 perc. pnts.  (vice-versa in brackets)  P2\_L: 39.0 (21.7)   P2prime\_L: 27.7 (13.9) |  |
| **P1\_Q**  15 in 163 sites   9.2 %    effects > 10 perc. pnts.  (vice-versa in brackets)  P3\_V: 18.6 (11.6)   P2\_S: 18.7 (21.6)   P2prime\_H: 10.8 (40.8)   P2prime\_Q: 13.3 (18.1) |  |
| **P1\_R**  18 in 163 sites   11.0 %    effects > 10 perc. pnts.  (vice-versa in brackets)  P2\_L: 11.2 (11.2)   P2prime\_I: 18.0 (20.2)   P3prime\_G: 27.2 (25.8) |  |
| **P1\_S**  14 in 163 sites   8.6 %    effects > 10 perc. pnts.  (vice-versa in brackets)  P2\_Q: 21.9 (27.8) |  |
